# The Optimization of Culture Conditions for Injectable Recombinant Collagen Hydrogel Preparation Using Machine Learning

**DOI:** 10.3390/gels11020141

**Published:** 2025-02-17

**Authors:** Mengyu Li, Long Zhao, Yanan Ren, Linfei Zuo, Ziyi Shen, Jiawei Wu

**Affiliations:** 1 Key Laboratory of Resource Biology and Modern Biotechnology in Western China, Ministry of Education, College of Life Sciences, Northwest University, Xi’an 710069, China; 2 Bytedance, Beijing 100034, China; 3 Provincial Key Laboratory of Biotechnology and Biochemical Engineering, School of Medicine, Northwest University, Xi’an 710069, China

**Keywords:** recombinant collagen, hydrogel, machine learning, decision tree, support vector machine, neural network

## Abstract

Injectable recombinant collagen hydrogels (RCHs) are crucial in biomedical applications. Culture conditions play an important role in the preparation of hydrogels. However, determining the characteristics of hydrogels under certain conditions and determining the optimal conditions swiftly still remain challenging tasks. In this study, a machine learning approach was introduced to explore the correlation between hydrogel characteristics and culture conditions and determine the optimal culture conditions. The study focused on four key factors as independent variables: initial substrate concentration, reaction temperature, pH level, and reaction time, while the dependent variable was the elastic modulus of the hydrogels. To analyze the impact of these factors on the elastic modulus, four mathematical models were employed, including multiple linear regression (ML), decision tree (DT), support vector machine (SVM), and neural network (NN). The theoretical outputs of NN were closest to the actual values. Therefore, NN proved to be the most suitable model. Subsequently, the optimal culture conditions were identified as a substrate concentration of 15% (*W*/*V*), a reaction temperature of 4 °C, a pH of 7.0, and a reaction time of 12 h. The hydrogels prepared under these specific conditions exhibited a predicted elastic modulus of 15,340 Pa, approaching that of natural elastic cartilage.

## 1. Introduction

Hydrogels are three-dimensional (3D) polymer networks that have been extensively utilized in tissue engineering, such as wound healing and tissue regeneration, due to their high water content and properties resembling the natural extracellular matrix (ECM), including biodegradability, porosity, and biocompatibility [1,2,3,4,5,6]. Moreover, hydrogels are capable of encapsulating and releasing small molecules, biomacromolecules, and cells, thereby finding applications in cancer immunotherapy [7]. In recent years, injectable hydrogels have emerged as a new hydrogel system with a certain degree of mobility, allowing them to be implanted into the body via injection [3,4,8,9,10,11].

Hydrogels are formed by hydrophilic homopolymers or copolymers through covalent bonds or physical or chemical attraction like crosslinking [12]. The polymers of hydrogels are categorized into two main types: natural polymers and synthetic polymers [13,14,15]. Natural polymers encompass polysaccharide-based polymers such as hyaluronic acid [16,17,18,19,20], agarose [21,22,23], chitosan [16,23,24,25,26,27,28,29,30,31,32,33], alginate [28,29,34,35,36,37,38,39], and heparin [19,40,41,42], as well as polyaminoacid-based polymers (proteins or peptides) like collagen [27,33,43,44,45,46,47], fibrin [28,48,49], gelatin [38,39,50], and elastin [51,52], and polynucleotide-based (DNA/RNA) polymers [7,15,53]. These three kinds of natural polymers can be combined to construct multifunctional hydrogels [54]. On the other hand, synthetic polymers like polyethylene glycol (PEG) [36,45,51,55,56,57,58,59,60,61], polycaprolactone (PCL) [46,62], polyurethane (PU) [18,63,64,65], polyvinyl alcohol (PVA) [30,66,67], polylactic acid (PLA) [31,32,68,69], and others are also utilized in hydrogel formation [15,53]. Furthermore, a combination of natural and synthetic polymers can be applied to fabricate hydrogels [18,30,31,32,36,45,46,66]. In the context of tissue engineering, hydrogels must fulfill several design criteria, including biodegradability, porosity, biocompatibility, cell adhesion, and other essential properties [15,70].

Collagen, as a kind of natural polymer, is the most predominant protein found in various human tissues such as cartilage, bones, skin, ligaments, and connective tissue, comprising one-third of the total protein content, and serves as an ideal material for preparing hydrogels [15,47,71,72]. Despite its abundance, natural collagen for tissue engineering scaffolds is difficult to extract, exhibits poor water solubility, and triggers strong immune responses [73,74]. Consequently, there has been a shift towards the artificial reorganization of collagen through gene engineering techniques, garnering considerable interest [75,76,77]. By integrating human collagen gene fragments into the Escherichia coli plasmid and carrying out fermentation purification, recombinant human-like collagen (HLC) can be synthesized by utilizing cutting-edge modern biotechnological approaches [33,77,78,79]. HLC, a product of gene engineering, not only replicates the properties of natural collagen but also possesses remarkable advantages such as low immunogenicity, ease of modification, absence of virus-hidden risks, high water solubility and has seen a surge in research and application in tissue filling and medical scaffolds [33].

The preparation process of recombinant collagen hydrogels (RCHs) is influenced by various factors such as the initial substrate concentration, reaction temperature, pH level, reaction time, crosslinking agent, and additives. However, understanding the combined impact of these factors on RCHs remains unclear and typically relies on experiments, lacking a reliable predictive model. To address this gap, mathematical models were introduced in this study. These models offer a means to obtain the characteristics of hydrogels under specific conditions without the need for experiments. Furthermore, they facilitate the optimization of culture conditions, thereby reducing both time consumption and material waste.

Traditional mathematical models include linear fitting, response surface methodology, and so on, which are employed to study the correlation between variables. However, with the expanding scale and complexity of data, traditional mathematical models have revealed limitations and machine learning has emerged as an increasingly reliable and particularly useful tool in addressing challenges [80].

Traditional machine learning models, not based on neural networks, such as decision trees (DTs) [81], random forests [81,82], extreme gradient boosting (XGBoost) [83,84], and support vector machines (SVMs) [85,86,87,88,89,90,91], are commonly used to address solve classification and regression problems [80]. In recent years, artificial neural network models, including multilayer perceptrons (MLPs) [92,93], convolutional neural networks (CNNs) [94,95,96], recurrent neural networks (RNNs) [97,98,99], graph convolutional networks (GCNs) [100,101,102,103,104], and so on, have rapidly developed, with them originally inspired by neurons in the brain. These models exhibit superior performance than traditional machine learning models in certain applications.

Training models can be executed by various software packages, including scikit-learn [105] and caret [106]. The scikit-learn software package, a machine learning method toolkit based on Python, is widely used for data preprocessing and model training within the field of data science. It can call a number of packages, thereby greatly simplifying the utilization of machine learning techniques and lowering the threshold for machine learning [105]. Here, multiple linear regression (MLP), decision tree (DT), support vector machine (SVM), and neural network (NN) were employed to investigate the correlation between hydrogel characteristics and culture conditions and determine the optimal culture conditions by using the scikit-learn module.

Machine learning models fundamentally operate on the principle of input and output. By utilizing a database consisting of independent variables (input) and dependent variables (output), a suitable model can be developed through training and testing processes. As the scale of the database increases, the precision of the model correspondingly improves. Consequently, after processing the input, the model generates the output. Therefore, machine learning is not only adaptable to various types of hydrogels and similar materials but also to a wide range of problems concerning input and output relationships.

In particular, this study focused on the impact of mechanical properties on cell growth and development, so the elastic modulus of hydrogels was selected to be the quantified feature. The elastic modulus, which is measured by a rheometer, represents the energy stored in a material due to elastic deformation and reflects the rigidity and solid characteristics of the material, serving as a crucial indicator for assessing the mechanical properties of biological tissues and biomaterials. Higher elastic modulus values indicate greater resistance to deformation. The mechanical properties of different organs determine tissue function and regulate cell behavior [107,108,109,110]. The elastic modulus varies significantly among different human tissues, playing a crucial role in cellular function performance. When constructing RCHs, the elastic modulus becomes particularly important for facilitating cell growth and differentiation, as well as ensuring the normal functioning of cells.

## 2. Results and Discussion

### 2.1. Single-Factor Experiment Analysis

**Figure 1:**
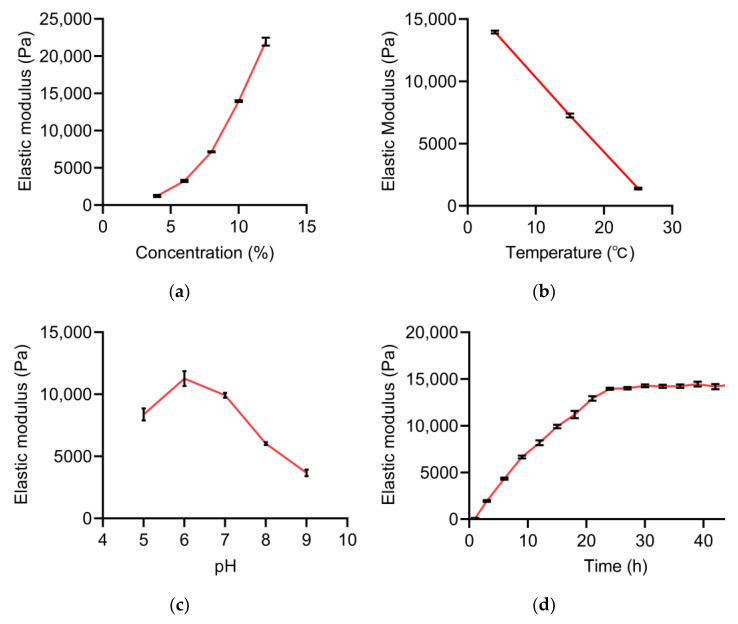
Analysis of the effect of a single factor on the elastic modulus of the hydrogels. Values represent the means ± SD. (**a**) Relationship between elastic modulus and substrate concentration (temperature = 4 °C, time = 24 h, pH = 7.0); (**b**) Relationship between elastic modulus and reaction temperature (concentration = 10%, time = 24 h, pH = 7.0); (**c**) Relationship between elastic modulus and pH (concentration = 10%, temperature = 4 °C, time = 15 h); (**d**) Relationship between elastic modulus and reaction time (concentration = 10%, temperature = 4 °C, pH = 7.0).

For each influencing factor, one set of data was selected to analyze its effect on the elastic modulus of the hydrogels (Figure 1). All 71 types of RCHs were prepared under varying concentrations, reaction temperatures, pH levels, and reaction times, and subsequently, the elastic modulus was measured (Table S1). In order to determine the impact of specific conditions on the elastic modulus, the average of three repeated experiments was utilized as the representative value.

The elastic modulus of the RCHs increased significantly with the increase in substrate concentration within the solubility range (Figure 1a). Specifically, at a concentration of 4%, the elastic modulus was measured at 1237 Pa. This value notably increased to 3255 Pa, representing a 160% increment, when the concentration was raised to 6%. The highest observed elastic modulus of 21,960 Pa was measured at a concentration of 12%. This trend demonstrated a clear relationship between substrate concentration and the resulting elastic modulus values of the RCHs.

Similarly, the elastic modulus of the RCHs was also influenced significantly by temperature variation, with a notable decrease observed as the temperature rose. As shown in Figure 1b, the highest elastic modulus of the RCHs was measured at 4 °C, reaching 13,963 Pa. Subsequent temperature increments to 15 °C and 25 °C resulted in a decrease in the elastic modulus to 7259 Pa and 1392 Pa, respectively, representing a substantial 48% and 90% reduction compared to the value at 4 °C. This decline was attributed to the impact of transglutaminase activity.

In addition to temperature, the activity of transglutaminase can be influenced by various pH levels. The elastic modulus of RCHs showed an interesting trend as the pH levels rose from 5 to 9. Specifically, the elastic modulus initially increased, reaching its peak at pH 6, and then declined thereafter (Figure 1c). At a pH of 6, the highest elastic modulus of the RCHs was measured at 11,275 Pa, indicating peak activity of transglutaminase at this point. However, as the pH exceeded 6, the elastic modulus decreased to 9921 Pa at pH 7, with a less statistically significant difference (*p*-value of 0.01) compared to pH 8 and 9, where the *p*-values were 5.99 × 10^−5^ and 1.82 × 10^−5^, respectively. Therefore, in our further study on obtaining the optimal conditions for hydrogel preparation, we selected a pH of 7 based on these findings.

Different from concentrations, temperatures, and pH levels, the cross-linking reaction of the RCHs progressed as time increased, eventually reaching a plateau phase. Initially, the elastic modulus of the RCHs showed a consistent increase, peaking at 13,963 Pa after 24 h (Figure 1d). It is noteworthy that the elastic modulus did not exhibit a further significant increase beyond this point. Therefore, in terms of time efficiency, it was recommended to finish the preparation of hydrogels after 24 h in the experimental process.

### 2.2. Model Analysis Results and Comparison

The training set in the database, comprising substrate concentration, reaction temperature, pH, and reaction time as independent variables along with the elastic modulus of the hydrogels as the dependent variable, was utilized. These variables served as inputs in the analysis conducted through the four aforementioned methods (ML, DT, SVM, and NN) to formulate mathematical models for hydrogel cross-linking.

#### 2.2.1. Multiple Linear Regression (ML)

The ML model, fitted by the LinearRegression in scikit-learn, yielded a score of 0.824 and a root mean squared error (RMSE) of 2164 on the test set (Table 1). Taking concentration (C), temperature (T_m_), pH (X), and time (T_i_) as four independent variables and the elastic modulus of the RCHs (G) as the dependent variable, this model generated the following multiple linear regression equation:

G = 845.720 C − 364.973 T_m_ − 925.119 X + 144.266 T_i_ + 6097.900

#### 2.2.2. Decision Tree (DT)

**Figure 2:**
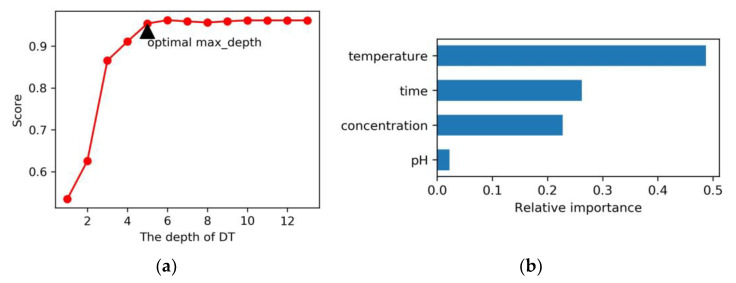
(**a**) The correlation between scores on the test set and max_depths of DT. The red dots represent the predicted scores corresponding to each depth level. The black triangle indicates that a depth of 5 represents the most suitable model; (**b**) The relative variable importance of four factors on the elastic modulus of DT.

The DT model was fitted by the DecisionTreeRegressor in scikit-learn. The adjustment of hyperparameters is an important issue in model training, significantly impacting model performance. For DT, the “max_depth” is a key hyperparameter to consider. To evaluate the relationship between the model’s score and the “max_depth” hyperparameter, a correlation plot was created (Figure 2a). The plot revealed that as max_depth increased, the mode’s score initially rose significantly, plateauing after reaching a value of 5. Hence, it was determined that an optimal max_depth of 5 is suitable for this DT model.

After investigating the training set samples, a decision tree with a depth of 5 was constructed (Figure S1). The root node, using all 49 training samples, was split at two levels of reaction temperature: ≤9.5 °C and >9.5 °C. The model achieved a score of 0.954 and a RMSE of 1108 on the test set (Table 1), indicating superior performance compared to ML. The impact of the independent variables on the elastic modulus varied, with different levels of importance. Utilizing the feature importance function (clf.feature_importances) of the DT model, the relative variable importance was quantified. Temperature, time, concentration, and pH accounted for 48.74%, 26.23%, 22.77%, and 2.26% of the overall importance, respectively (Figure 2b).

#### 2.2.3. Support Vector Machine (SVM)

The SVM model in scikit-learn was fitted using SVR. Before fitting, three critical hyperparameters needed to be set for SVM: “kernel functions”, “C”, and “gamma”. Kernel functions aid in solving nonlinear problems by transforming high-dimensional space. The regularization parameter C governs the complexity of the model. A larger C results in a tighter decision margin and a more intricate model and potentially leads to overfitting. Meanwhile, the kernel coefficient gamma determines the influence of a training data point on the hyperplane. A higher gamma increases the impact of a training data point on the hyperplane, making it more likely to be selected as a support vector. Much like C, an elevated gamma value may also lead to overfitting.

**Figure 3:**
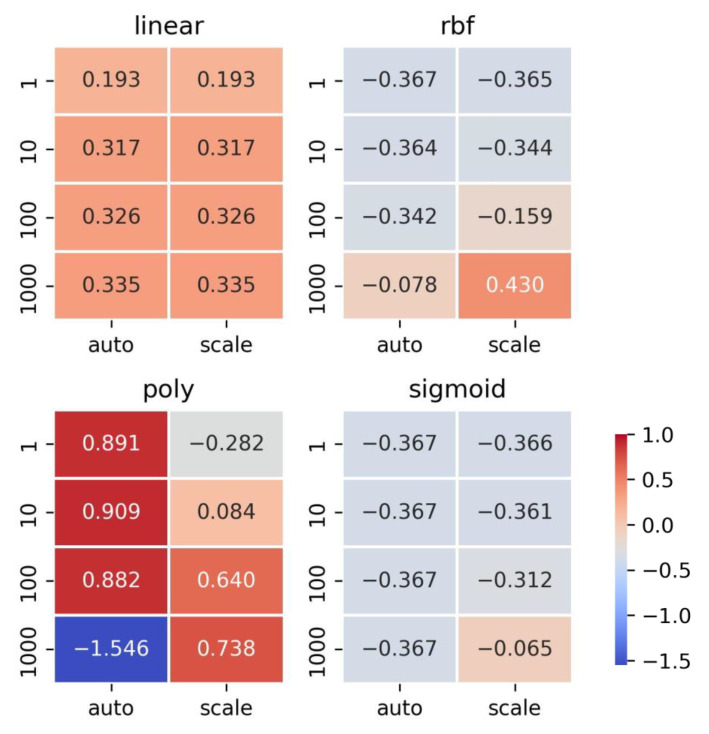
Cross-validation and grid search results of SVM. The heatmap cell values represent the scores predicted by the model.

In the following SVM model, cross-validation and grid search were employed to tune those three hyperparameters using GridSearchCV. A total of 32 models were created, each incorporating different combinations of four kernel functions (“linear”, “rbf”, “poly”, and “sigmoid”), four C values (1, 10, 100, and 1000), and two gamma values (“auto” and “scale”) (Figure 3). Among these combinations, the best-performing hyperparameters were found to be kernel = ‘poly’, C = 10, and gamma = ‘auto’, achieving the highest score of 0.909. Upon training the model with the complete training set, it yielded an impressive score of 0.980 and a RMSE of 714 (Table 1). Subsequently, when tested on the test set, the model demonstrated a score of 0.985 and a RMSE of 625 (Table 1), indicating high accuracy levels without overfitting.

#### 2.2.4. Neural Network (NN)

Because natural language processing and figure recognition were not required, the easiest choice for analyzing mathematical data was to use the multilayer perceptron (MLP). In this study, the model was fitted using the MLPRegressor in scikit-learn. Comparing MLP with DT and SVM, it was important to note the numerous hyperparameters involved in the fitting process.

The first critical hyperparameter to consider was “activation function”. Popular activation functions, such as Sigmoid, Tanh, and ReLU (Rectified Linear Unit), are commonly used in neural networks. Both Sigmoid and Tanh functions are effective in handling binary or quasi-binary problems, but they are susceptible to issues like gradient disappearance or explosion. On the other hand, ReLU offers better stability and computational efficiency and is widely adopted in deep learning approaches. As a result, ReLU was chosen as the activation function to fit the model.

The second hyperparameter to consider was “learning_rate_init”. A smaller learning_rate_init leads to a slower study process and longer model fitting time. When using the default value of 0.001 for learning_rate_init, a warning message arose: “Stochastic Optimizer: Maximum iterations reached and the optimization hasn’t converged yet”, even when a large maximum number of iterations (“max_iter”) was set. To address this issue, a value of 0.01 was selected for learning_rate_init, along with the implementation of 3-5 hidden layers of neurons. This adjustment was made to enhance the convergence of the optimization process and improve the efficiency of model training.

**Figure 4:**
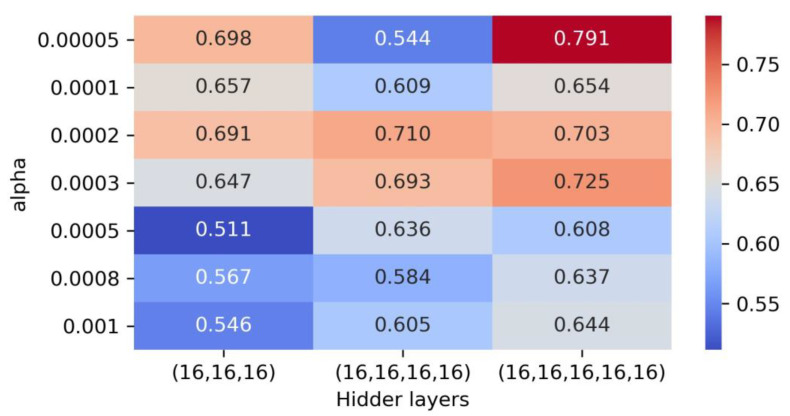
Cross-validation and grid search results of NN. The heatmap cell values represent the scores predicted by the model.

Finally, in order to prevent overfitting, it was crucial to introduce the hyperparameter “alpha”. Here, different alpha values ranging from 0.00005 to 0.001, including 0.00005, 0.0001, 0.0002, 0.0003, 0.0005, 0.0008, and 0.001, were deliberated upon. Through cross-validation and grid search, the analysis identified alpha as 0.00005, coupled with “hidden_layer_sizes” of (16, 16, 16, 16, 16), presenting the best outcome with a notable score of 0.791 (Figure 4). Upon training the entire training set holistically, a substantially higher score of 0.996 and a correspondingly lower RMSE of 308 were obtained, surpassing the performance of the SVM model and with the potential presence of overfitting. Subsequently, the evaluation of the model’s performance on the test set reflected a score of 0.985 and a RMSE of 623, approximately two times greater than that observed in the training set, corroborating the existence of overfitting. Consequently, attention was focused on the second-ranking model, which showcased a score of 0.725, when alpha increased to 0.0003 (Figure 4). Following the total fitting of the training data, this model attained a score of 0.991 and a RMSE of 468 on the training set. Notably, the performance evaluation on the test set indicated a significantly improved score of 0.992 and a RMSE of 469 (Table 1), exhibiting superior performance when compared to the SVM model, devoid of evident overfitting concerns.

### 2.3. Model Evaluation and Prediction of Optimal Conditions

#### 2.3.1. Model Evaluation

**Figure 5:**
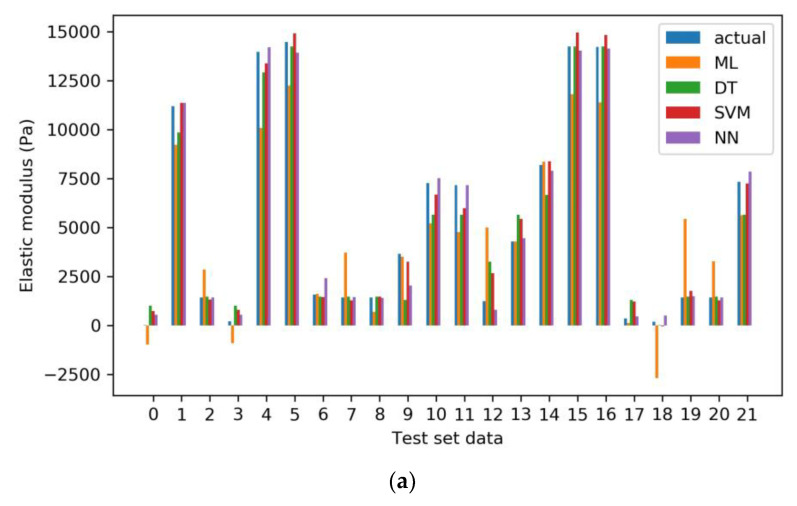

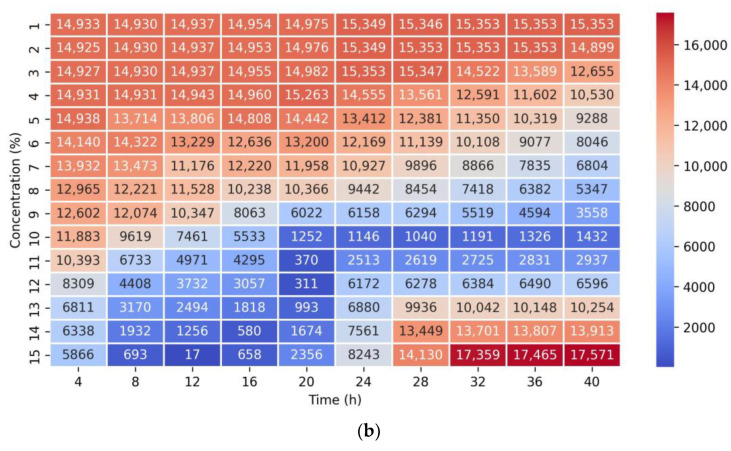
(**a**) The comparison between theoretical outputs and actual values for each of the four models; (**b**) The effects of time and concentration on the elastic modulus in optimizing the preparation process of RCHs. The heatmap cell values represent the absolute difference between the predicted values under specific conditions and the elastic modulus of natural elastic cartilage, which is 15,357 Pa.

In order to evaluate the predictive accuracy of the four models above, the theoretical values of the test set were calculated for each of the four models mentioned above: ML, DT, SVM, and NN. Subsequently, the theoretical outputs were compared with the actual values for each model. The analysis revealed that NN demonstrated the highest level of predictive accuracy, closely matching the actual values. Following NN, SVM displayed relatively strong predictive performance. In contrast, the calculated values for DT showed a larger deviation from the actual values compared to NN and SVM. Lastly, ML exhibited the largest deviation between its theoretical and actual output values (Figure 5a).

The RMSEs of the ML, DT, SVM, and NN models were calculated to assess the deviation between the predicted and observed values on the test set. The RMSE values for ML, DT, SVM, and NN were found to be 2164, 1108, 625, and 469, respectively (Table 1). Notably, the RMSE of the NN model was the lowest among all models, suggesting that its predictions closely matched the actual values. Moreover, the scores for the ML, DT, SVM, and NN models on the test set were 0.824, 0.954, 0.985, and 0.992, respectively (Table 1), indicating that the NN model performed best.

These findings aligned with the results illustrated in Figure 5a, thus indicating the superior predictive performance of the NN model in this analysis.

Herein, the NN model was chosen as the optimal mathematical model for analyzing the elastic modulus of RCHs prepared under various cross-linking conditions. By utilizing this model, it is possible to predict the elastic modulus of hydrogels under specific culture conditions, obtain the optimal culture condition, and determine the necessary range of experimental conditions for preparing RCHs based on specific mechanical property requirements, thereby reducing both the workload and associated economic costs.

#### 2.3.2. Prediction of Optimal Conditions

To optimize the preparation process of RCHs, the temperature and pH were fixed at 4 °C and 7, respectively, considering protein and cell activities (Figure 1b,c). The effects of time and concentration on the elastic modulus were then analyzed. The values of the “time” variable set varied from 4 to 40 h (at intervals of 4 h), while the “concentration” variable set ranged from 1% to 15% (at intervals of 1%). Subsequently, data from 150 groups were input into the NN model to predict the elastic modulus, with the outcomes depicted in Figure 5b (Table S2).

The elastic modulus of natural elastic cartilage was found to be 153,57 ± 133.0 Pa. Notably, the elastic modulus of the RCHs under one of the specific conditions most closely approximated that of natural elastic cartilage, indicated by a reaction time of 12 h with a substrate concentration of 15%. Under this optimized condition, the predicted elastic modulus of the hydrogels was 15,340 Pa, approaching that of natural elastic cartilage. The prediction is promising for cartilage regeneration and repair. However, the actual impact of RCHs prepared under these conditions on seed cell growth and cartilage repair remains to be explored. Future research should focus on these aspects to better understand the potential of RCHs in clinical applications within the field of cartilage regeneration and repair engineering.

## 3. Conclusions

The establishment of culture conditions and hydrogel characteristics during the preparation of RCHs is a complex issue. This work proposed ML, DT, SVM, and NN to establish mathematical models for hydrogel cross-linking. Among these models, NN yielded the highest score and the smallest RMSE, and output the prediction results wich were the closest to the actual values. Therefore, it was identified as the most suitable model and subsequently utilized to predict the optimal conditions for preparing the RCHs. Under that optimized condition, the predicted elastic modulus of the RCHs was 15,340 Pa. It is possible that optimized RCHs facilitate seed cell growth and could be applied in cartilage regeneration and repair.

The successful application of NN in predicting these conditions offers a dependable method for the production of various hydrogels. Nonetheless, further research is needed to explore the characteristics of RCHs prepared under optimal conditions, such as their impact on seed cell growth and cartilage regeneration. These investigations will contribute to advancing clinical research in the field of cartilage regeneration and repair engineering.

In the training process of NN, overfitting is a prominent issue that readily arises. For instance, when fitting the network with 3–5 hidden layers, each comprising 16 neurons and using a learning rate of 0.001, the fitting score reached 1, indicating clear overfitting and rendering the results unreliable. As a remedy, it is recommended to increase the initial learning rate. Furthermore, it is advisable to incorporate the L2 regularization technique to enhance the model’s generalization capabilities.

The comparison of NN and SVM performance revealed that NN yielded superior results, while SVM lagged slightly behind. However, both models have their respective advantages and disadvantages. Notably, the hyperparameters of NN are significantly more numerous compared to SVM, with SVM exhibiting slower computational speed. Following the determination of hyperparameters, SVM required 18 s for model fitting and prediction, in contrast with NN’s instantaneous performance. The computational burden and duration of running cross-validation and grid search are more pronounced, implying that SVM consumes greater memory resources. Thus, the decision on whether to utilize SVM or NN should be contingent upon the specific requirements of the task at hand.

## 4. Materials and Methods

### 4.1. Single-Factor Experiments

The independent variables selected for this study were the initial substrate concentration, reaction temperature, pH level, and reaction time, with the elastic modulus of hydrogels as the dependent variable. Prior to experimentation, suitable ranges for each independent variable were established. In the experiments, 0.01 g of transglutaminase (Shanghai yuanye Bio-Technology Co., Ltd, Shanghai, China) was first dispensed into a 1 mL reaction system (20 U/g), followed by the addition of the cross-linking reaction substrate Recombinant Collagen (RC) at varying concentrations (*W*/*V*). The effects of different concentrations (4%, 6%, 8%, 10%, 12%, and 15%), reaction temperatures (4 °C, 15 °C, and 25 °C), pH levels (5, 6, 7, 8, and 9), and reaction times (0.5 h, 1 h, 2 h, 3 h, 4 h, 5 h, 6 h, 9 h, 12 h, 15 h, 18 h, 21 h, 24 h, 27 h, 30 h, 33 h, 36 h, 39 h, 42 h, 45 h, and 48 h) on the elastic modulus were then investigated.

Each single-factor experiment was conducted three times using various combined conditions to prepare the RCHs. Subsequently, three cylinders measuring 20 mm in diameter and 1 mm in height were created for testing.

### 4.2. Elastic Modulus Detection

A rheometer (DHR-2, Waters Corp., Milford, Massachusetts, USA) was used to measure the elastic modulus of the RCHs at a temperature of 20 °C, a frequency of 1 Hz, and a strain range of 0.1–100 Pa. 

### 4.3. Model Fitting and Data Analysis

The Single-factor experimental data were analyzed using GraphPad Prism 9.0 software (GraphPad Software Inc., San Diego, California, USA). Statistical significance testing comparing the various RCHs was conducted using the F-test and *t*-test. 

The data obtained from all experimental tests were used to create a database, which was subsequently randomly split into two subsets: a training set (70%) and a test set (30%). The training set was employed to train and fit the proposed mathematical model using four algorithms: ML, DT, SVM, and NN. Following this, the test set was utilized to assess the model’s validity. The implementation of the four models was carried out using scikit-learn (https://scikit-learn.org/stable/ (accessed on 9 April 2024)) in Python. The graphs were plotted using matplotlib (https://matplotlib.org (accessed on 12 January 2025)) and seaborn (https://seaborn.pydata.org (accessed on 12 January 2025)) in Python.

## Figures and Tables

**Table 1:** Comparison between scores and RMSEs for each of the four models.

Model	Test Set		Training Set	
	Score	RMSE	Score	RMSE
ML	0.824	2164	0.672	2872
DT	0.954	1108	0.977	757
SVM	0.985	625	0.980	714
NN	0.992	469	0.991	468

## Data Availability

The original contributions presented in this study are included in the article/Supplementary Material. Further inquiries can be directed to the corresponding author.

## Supplementary Materials

The following supporting information can be downloaded at: https://www.mdpi.com/article/10.3390/gels11020141/s1. Figure S1: The decision tree with a depth of 5; Table S1: The elastic modulus of all 71 types of RCHs; Table S2: The effects of time and concentration on the elastic modulus in optimizing the preparation process of RCHs.
