# Supplementary figures and images for: The Optimization of Culture Conditions for Injectable Recombinant Collagen Hydrogel Preparation Using Machine Learning
Source: Gels. 2025 Feb 17;11(2):141. doi: 10.3390/gels11020141 (PMC11855032; doi:10.3390/gels11020141)

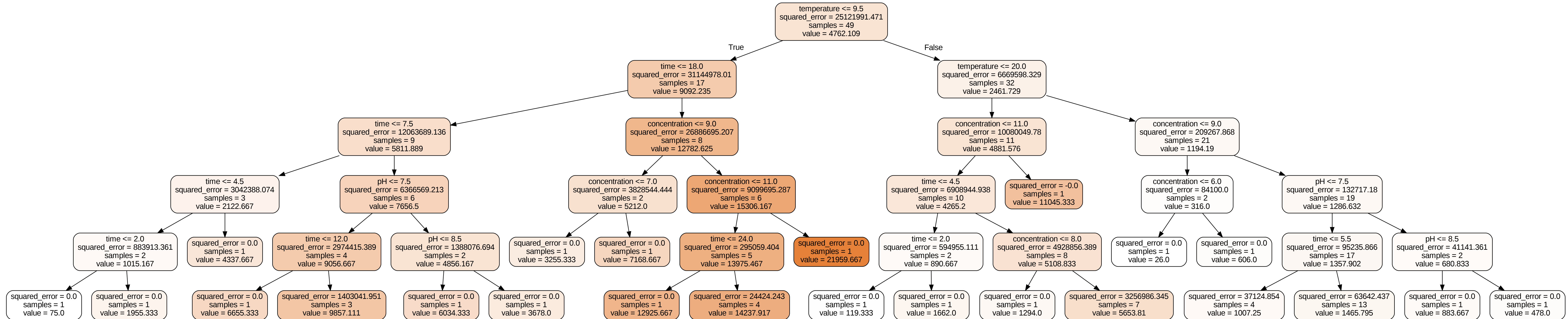

Supplement: Supplementary file 1 [file gels-11-00141-s001.zip › Figure S1.pdf]
